# Altered oscillation patterns and connectivity during picture naming in autism

**DOI:** 10.3389/fnhum.2013.00742

**Published:** 2013-11-08

**Authors:** Isabelle Buard, Sally J. Rogers, Susan Hepburn, Eugene Kronberg, Donald C. Rojas

**Affiliations:** ^1^UCD Magnetoencephalography Lab, Department of Psychiatry, University of Colorado at Denver – Anschutz Medical CampusAurora, CO, USA; ^2^Psychiatry and Behavioral Sciences, UC Davis MIND InstituteSacramento, CA, USA; ^3^University of Colorado/JFK PartnersAurora, CO, USA

**Keywords:** magnetoencephalography, gamma-band, beta-band, oscillations, functional connectivity, Granger causality, fusiform gyrus, endophenotype

## Abstract

Similar behavioral deficits are shared between individuals with autism spectrum disorders (ASD) and their first-degree relatives, such as impaired face memory, object recognition, and some language aspects. Functional neuroimaging studies have reported abnormalities in ASD in at least one brain area implicated in those functions, the fusiform gyrus (FG). High frequency oscillations have also been described as abnormal in ASD in a separate line of research. The present study examined whether low- and high-frequency oscillatory power, localized in part to FG and other language-related regions, differs in ASD subjects and first-degree relatives. Twelve individuals with ASD, 16 parents of children with ASD, and 35 healthy controls participated in a picture-naming task using magnetoencephalography (MEG) to assess oscillatory power and connectivity. Relative to controls, we observed reduced evoked high-gamma activity in the right superior temporal gyrus (STG) and reduced high-beta/low-gamma evoked power in the left inferior frontal gyrus (IFG) in the ASD group. Finally, reductions in phase-locked beta-band were also seen in the ASD group relative to controls, especially in the occipital lobes (OCC). First degree relatives, in contrast, exhibited higher high-gamma band power in the left STG compared with controls, as well as increased high-beta/low-gamma evoked power in the left FG. In the left hemisphere, beta- and gamma-band functional connectivity between the IFG and FG and between STG and OCC were higher in the autism group than in controls. This suggests that, contrary to what has been previously described, reduced connectivity is not observed across all scales of observation in autism. The lack of behavioral correlation for the findings warrants some caution in interpreting the relevance of such changes for language function in ASD. Our findings in parents implicates the gamma- and beta-band ranges as potential compensatory phenomena in autism relatives.

## INTRODUCTION

High-frequency brain activities have a central role in various normal functions (Buzsaki and Draguhn, 2004), including sensory binding (Rodriguez et al., 1999), temporal regulation of neuronal activity during synaptic plasticity (Traub et al., 1998), memory processing (Fell et al., 2001), and large-scale integration (Varela et al., 2001). Several suggestions have been proposed to define the role of gamma-band oscillations (30 Hz and higher) as a correlate of auditory awareness (Makeig and Jung, 1996; Yordanova et al., 2002) or encoding mental representations (Tallon-Baudry and Bertrand, 1999). Moreover, the correlation between gamma synchronization and hemodynamic responses reconciles common findings in fMRI and brain electrophysiology (Niessing et al., 2005). Particularly, gamma-band has been associated with face processing, notably in the fusiform gyrus (FG; Zion-Golumbic and Bentin, 2007; Gao et al., 2013). In autism spectrum disorders (ASD), impairments of gamma oscillations have been previously described in auditory (Wilson et al., 2007; Gandal et al., 2010) and visual domains (Grice et al., 2001; Brown et al., 2005; Milne et al., 2009; Isler et al., 2010; Stroganova et al., 2012), suggesting a link between high-frequency oscillations and perceptual dysfunction. We have also established that these deficits are seen in adult first-degree relatives, suggesting that such impairment constitutes an autism endophenotype (Rojas et al., 2008, 2011; McFadden et al., 2012). Lower frequency oscillatory activity has also been described as affected in autism, such as impaired mu wave suppression during action observation (Oberman et al., 2005).

Autism is defined by a triad of core impairments in social interaction, communication, and behavioral flexibility (American Psychiatric Association, 2000). Communication deficits include individual with autism’s difficulty using spoken language and gestures, inability to initiate and sustain appropriate conversation and use of inappropriate, repetitive language (Lord et al., 2000). The severity of language impairment is highly variable in autism, ranging from highly verbal to essentially non-verbal (Tager-Flusberg et al., 2009), and it remains the best-known indicator of prognosis in affected individuals (Venter et al., 1992). Within the language domain, problematic pragmatic language use has been repeatedly documented among relatives (Losh et al., 2008). Among language impairments, word processing is particularly affected in ASD (Walenski et al., 2008). To examine the prediction of altered lexical processing, we tested subjects with autism on a picture-naming task, in which subjects named pictures of objects. Two major areas in the human brain are responsible for language (Binder et al., 1997): Broca’s area (localized to left inferior frontal gyrus, or IFG) which is involved in language production, and Wernicke’s area (localized in the superior temporal gyrus, STG) which is thought to be implicated in language processing. Other brain structures may also play a role in language. Among them, the FG has been initially studied as being a part of the visual system specialized in facial recognition (Fusiform Face Area; Kanwisher et al., 1997) because of the importance of face processing to successful social functioning. Its additional role in language processing, called the visual word form area (McCandliss et al., 2003), highlights its relevance for language studies. Interestingly, individuals with ASDs show atypical functional lateralization, with reduced left hemisphere and/or reversed patterns of cortical activation in linguistic experiments (Just et al., 2004; Flagg et al., 2005; Wang et al., 2006; Frye and Beauchamp, 2009).

Hypoactivity in the FG (Schultz et al., 2000) and IFG (Groen et al., 2010) areas has been reported in individuals with autism, suggesting that there should be physiological signatures underlying autism-related language impairments. The objective of this study was to compare gamma-band oscillations in the FG, STG, and IFG, language-related areas of control participants to patients with autism and first-degree relative of persons with ASD during a picture-naming task. Based on prior findings from simple auditory and visual processing experiments, as well as face perception experiments, we expected to observe reduced phase-locked, or evoked gamma-band activity in both the autism group and in parents of individuals with autism compared to controls. Increases in non-phase-locked, or induced gamma-band activity have also been reported in autism (e.g., see Brown et al., 2005; Rojas et al., 2008). We therefore separately analyzed the evoked and induced gamma-band activity in the study.

Building upon previous studies, we found some differential activation in the gamma- and beta-band range in people with autism compared to their first-degree relatives. Patterns of activation were opposite, as parent brains were over-activated while autistic brains showed under-activation. The connectivity analyses and results add to the existing literature by extension to an object naming task and examination of both individuals with autism and first-degree relatives.

## MATERIALS AND METHODS

### SUBJECTS

Participants were 12 persons with ASD, 16 parents of a child with ASD (PASD), and 35 controls (**Table 1**). One-way ANOVAs were used to examine demographic variables (age) for significant differences. No significant group differences were present at *p* > 0.05 for any of these group characteristics. For ASD subjects, diagnosis was based on convergence of clinical judgment by experimenters using DSM-IV criteria (American Psychiatric Association, 2000), and research reliability trained on the Autism Diagnostic Interview, Revised (ADI-R; Lord et al., 1994), and the Autism Diagnostic Observation Schedule (ADOS; Lord et al., 2000). Each of the 16 PASD group subjects had a single child who met the same criteria for ASD as the ASD group participants. The PASD group subjects were not biologically related to the study participants in the ASD group. The healthy comparison subjects had no personal history of developmental, psychiatric, or neurologic disorders, and no family history of developmental disorders. All subjects signed informed consent to participate in the study consistent with the guidelines of the Colorado Multiple Institution Review Board.

**Table 1:** Participants characteristics.

Measure	Controls	ASD	Parents	*p*-value
Age (mean years ± SD)	34.2 ± 11.9	28.3 ± 13.3	37.9 ± 5.9	0.082
Handedness score	0.62 ± 0.53	0.35 ± 0.79	0.74 ± 0.41	0.202

Handedness was assessed in all subjects using the Annett Handedness Questionnaire (Annett, 1985). Handedness score means were 0.62 ± 0.53, 0.35 ± 0.79, and 0.74 ± 0.41 for healthy controls, ASD subjects and PASD, respectively (**Table 1**). One-way ANOVA (SPSS version 21 – IBM Corp, Armonk, NY, USA) revealed no difference among groups: *F*(2, 54) = 1.65, *p* = 0.202.

### STIMULI AND EXPERIMENTAL DESIGN

The stimuli consisted of 192 black and white line art images from the International Picture Naming Project database^1^, which includes items from the Peabody Picture Vocabulary Test (PPVT; Dunn, 1997), Snodgrass and Vanderwart (1980) and other sources. The pictures represent simple objects such as a shovel or an airplane. Trials consisted of periods of picture stimuli lasting for 1200 ms, followed by a central fixation cross for a random inter-stimulus interval between 3000 and 5000 ms. Picture stimuli were presented by an LCD projector onto a screen located 45 cm in front of the subject and subtended an average of 7.27° horizontal visual angle and 6.02° vertical visual angle. Subjects were instructed to sub-vocalize (whisper) the name of the object depicted in the image they had just seen as soon as the fixation cross appeared (i.e., after the picture was removed) and received practice trials until they understood the instructions. Sub-vocalization was used instead of overt naming to reduce motion and muscle artifact in the MEG data. The entire recording session lasted approximately 16 min.

### MEG DATA ACQUISITION AND PRE-PROCESSING

We employed a Magnes 3600 WH whole-head MEG device (4-D Neuroimaging, San Diego, CA, USA), which comprises 248 first-order axial-gradiometer sensors in a helmet-shaped array. Five head position indicator coils attached to the subject’s scalp were used to determine the head position with respect to the sensor array. The locations of the coils with respect to three anatomical landmarks (nasion and pre-auricular points) and two extra non-fiducial points, as well as the scalp surface were determined with a 3D digitizer (Polhemus, Colchester, VT, USA). Identifying the three fiducial points on an SPM standard head model established the coordinate transformation between MEG and the standard MRI template used for the volume conductor in source modeling.

The MEG signals were acquired in a 0.1–200 Hz bandwidth and sampled continuously at 508 Hz and 24-bit quantization. MEG data pre-processing was conducted using the 4-D Neuroimaging software, Fieldtrip^2^ and Statistical Parametric Mapping SPM8 (Wellcome Trust Centre for Neuroimaging, London, UK) implemented in Matlab (2009b; MathWorks, Inc., Natick, MA, USA). Eye movement and blink artifacts were corrected using independent components analysis using the FastICA algorithm (Hyvarinen, 1999). Epochs were then defined of 1200 ms duration, with a baseline of -200 to 0 ms pre-stimulus onset and 1000 ms post-stimulus. Epochs were baseline corrected to remove any DC offset and those trials contaminated by excessively large MEG amplitudes (±3000 fT) were rejected from further analysis. An average of 119 (±25) artifact free epochs was obtained for source analysis.

### MEG SOURCE ANALYSIS AND SOURCE SPACE STATISTICS

Source analysis was performed in Matlab (2009b; MathWorks, Inc., Natick, MA, USA) using the SPM8 toolbox (Statistical Parametric Mapping; Wellcome Department of Cognitive Neurology, London, UK). Following co-registration of the MEG fiducials with the SPM8 standard template, leadfields were computed using a single shell volume conductor model. Source localization was then performed using a cortically constrained group minimum norm inversion with multiple sparse priors (Litvak et al., 2011), on all subjects’ data pooled together from the three groups, which resulted in a common source space images across subjects. The cortical surface used was a standard MNI space surface with 20484 vertices supplied within SPM8. Source analysis was performed on the 35–120 Hz passband between 100 and 250 ms.

Source space images were submitted to GLM-based statistical analysis using a one-sample *t*-test across subjects in all three groups to find a common set of activated regions for subsequent spectral analyses. Several active brain regions were obtained (**Table 2**), where activity during the task survived multiple comparison correction, using a false discovery rate (FDR) of *q* < 0.05. Among all active regions, we focused on the FG, the inferior frontal gyrus, the STG, and the occipital lobe (OCC) for further ROI-based analyses for three reasons: (1) their relevance to language function, (2) the engagement of visual structures in this specific task, and (3) leadfield correlation is high among closely spaced regions and induces artificial correlation in source waveforms derived from such locations. A limited set of widely spaced ROI is therefore more appropriate given these correlations.

**Table 2:** List of brain regions that were significantly active (FDR *q* < 0.05) from source analyses of the 35–120 Hz band between 100 and 250 ms post-stimulus.

Region	Side	MNI coordinates			*T* value
Pre-cuneus	R	4	-70	36	32.78
	L	-7	-74	42	32.12
Cuneus	R	10	76	38	32.62
	L	-10	-68	42	31.25
Inferior temporal gyrus area (includes **fusiform gyrus**)	R	54	-34	-26	11.18
	L	-48	-56	-14	18.78
Pre-central gyrus	R	42	-14	48	15.81
Post-central gyrus	R	44	-26	46	13.96
**Superior temporal gyrus**	R	50	-44	16	11.18
	L	-50	-44	18	16.92
Basal forebrain	R	22	10	-18	12.87
	L	-24	8	-18	12.87
**Inferior frontal gyrus**	R	42	28	16	12.18
	L	-42	24	18	12.24
Inferior frontal orbital	L	-34	40	-14	9.71
**Lobe occipital Superior**	R	22	-80	40	26.90
	L	-18	-80	34	25.88
Superior frontal gyrus	R	20	-8	64	6.69
	L	-20	-6	66	6.70

*Regions in bold print were selected for ROI based analyses.*

### SOURCE WAVEFORMS, SPECTRAL ANALYSES, AND FUNCTIONAL CONNECTIVITY

Regional time-courses were created via source-space projection (Tesche et al., 1995) from dipoles within each region of interest: left and right FG, IFG, STG, and OCC. We computed the lead field and its pseudoinverse and then we created current source waveform (Ross et al., 2000). Montreal Neurological Institute (MNI) coordinates described in **Table 2** were used for this step. Afterward, from those source-space projections were computed time-frequency transforms using a Morlet wavelet decomposition with wave number linearly increasing from 3 to 12 across the frequency range of 10–110 Hz, on the epochs from -200 to 800 ms. For each subject, evoked and induced power, relative to the 200 ms pre-stimulus baseline, were calculated, along with the phase-locking factor (PLF; Tallon-Baudry et al., 1996), a measure of inter-trial phase-consistency (also sometimes referred to as intertrial coherence). Mass univariate, non-parametric statistical analyses were performed across the entire time-frequency space, corrected for multiple comparisons using cluster size metrics at FWE < 0.01. Fieldtrip’s cluster-based correction for multiple comparisons uses Monte Carlo randomization to compute a sampling distribution for cluster sizes. Our threshold for cluster formation was set to *p* = 0.01 and the number of permutations set to 1000 (Maris and Oostenveld, 2007).

In order to evaluate directional functional connectivity between our regions of interest in the frequency domain, we computed frequency domain Granger causality using the Fieldtrip connectivity analysis functions (Oostenveld et al., 2011), which first involved an autoregressive model fit to the data using the bsmart matlab toolbox (Cui et al., 2008). For these analyses, we downsampled the data to 250 Hz for better model order estimation and submit the data to detrenting, differencing, and pre-whitening. Then, we estimated the model order to be 15 (60 ms) using ARfit toolbox for Matlab (Schneider and Neumaier, 2001). Group comparisons of Granger spectra were analyzed between regions of interest and between the two hemispheres, and corrected for multiple comparisons using the FDR method on the overall set of comparisons, *q* < 0.1.

### BEHAVIORAL TESTING

Since we did not measure spoken responses due to concern over excessive motion, the PPVT was performed and scored independently outside of the scanner, on a separate day following the MEG session, as a proxy indication of picture naming performance. The 192 items presented in the scanner contained 62 pictures from the PPVT items. A Kruskal–Wallis one-way analysis of variance was applied to test for statistical differences between groups.

## RESULTS

### SOURCE ANALYSIS RESULTS

**FIGURE 1:**
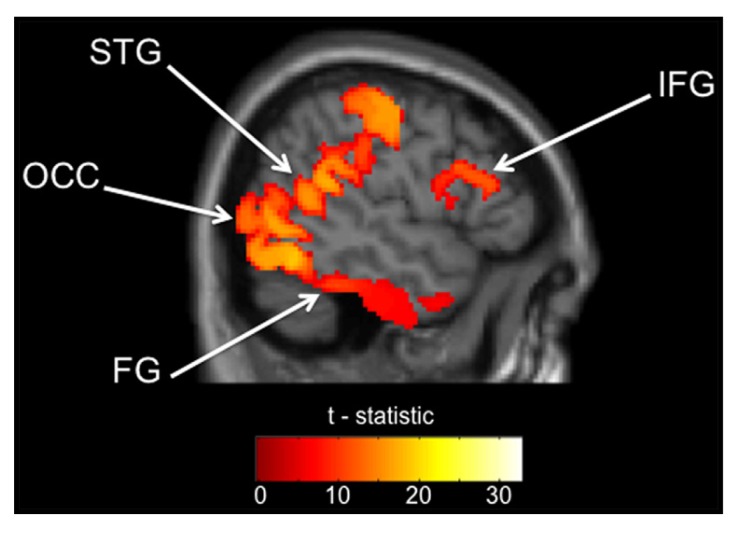
**Regions of interest from within source analysis results.** Gamma (35–120 Hz) activation maps 100–250 ms after stimulus presentation (FWE, *p* < 0.05) across all participants. FG, fusiform gyrus; STG, superior temporal gyrus; IFG, inferior frontal gyrus; OCC, occipital lobe. All four clusters used to define our ROIs are depicted in this slice, but the exact location of the MNI coordinate used is listed in **Table 2**.

**Table 2** presents the regions that were significantly active from the source analyses of the 35–120 Hz band between 100 and 250 ms. Indeed, those brain regions included mainly visual and language areas. Among them, the four cortical regions of interest to us during this task (FG, STG, IFG, and OCC) that were selected for source space projections and time-frequency analyses are depicted in **Figure 1.**

### TIME-FREQUENCY RESULTS

#### Fusiform gyrus

**FIGURE 2:**
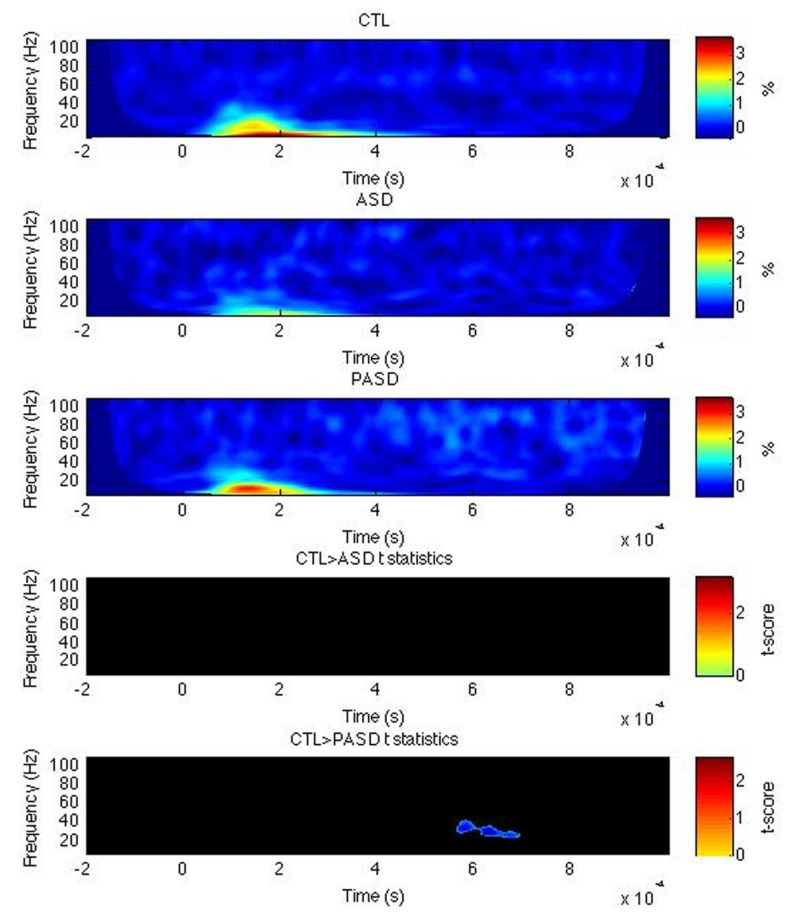
**Fusiform gyrus evoked power time-frequency results.** Grand average evoked power is shown for each group (three top rows) for the left fusiform gyrus (FG). *T*-statistic maps are shown in the two bottom rows that illustrate contrasts between controls (CTL) and ASD (row 4) and CTL and parents (PASD; row 5). Masked *t*-score results represent cluster-corrected findings at *p* < 0.01.

For the left FG, the evoked power was significantly higher in the PASD group, relative to the controls, for high beta/low gamma-band activity centered around 35 Hz and from around 580–700 ms post-stimulus onset (**Figure 2**). No differences in PLF or induced power were observed for the FG. No difference between the ASD group and both other ones were found in either left or right FG.

#### Superior temporal gyrus

**FIGURE 3:**
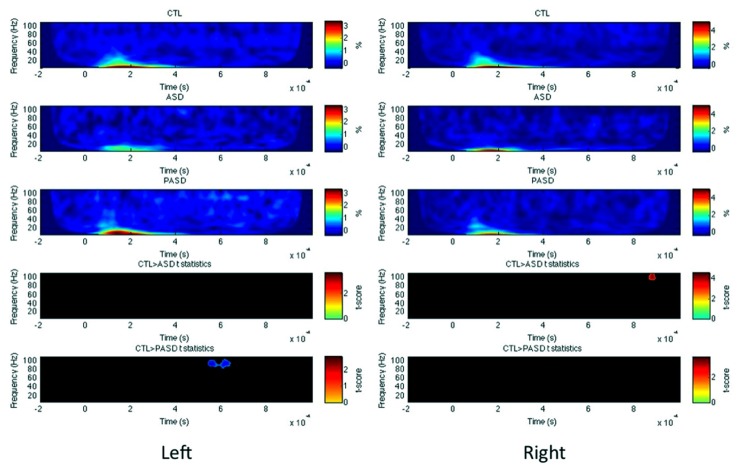
**Superior temporal gyrus evoked power time-frequency results.** Grand average evoked power is shown for each group (three top rows) for the left and right superior temporal gyrus (STG). *T*-statistic maps are shown in the two bottom rows that illustrate contrasts between controls (CTL) and ASD (row 4) and CTL and parents (PASD; row 5). Masked *t*-score results represent cluster-corrected findings at *p* < 0.01.

In the left STG, no significant differences were observed between the HC and ASD groups, but there was a significant increase in high-gamma evoked power for the PASD group relative to controls between 570 and 630 ms post-stimulus (**Figure 3**). No differences in PLF or induced power were seen for the left STG. In the right hemisphere STG, there was a significant decrease in high-gamma evoked power peaking at 900 ms in the ASD group compared to controls. No differences in PLF or induced power were observed in the right STG between groups.

#### Inferior frontal gyrus

**FIGURE 4:**
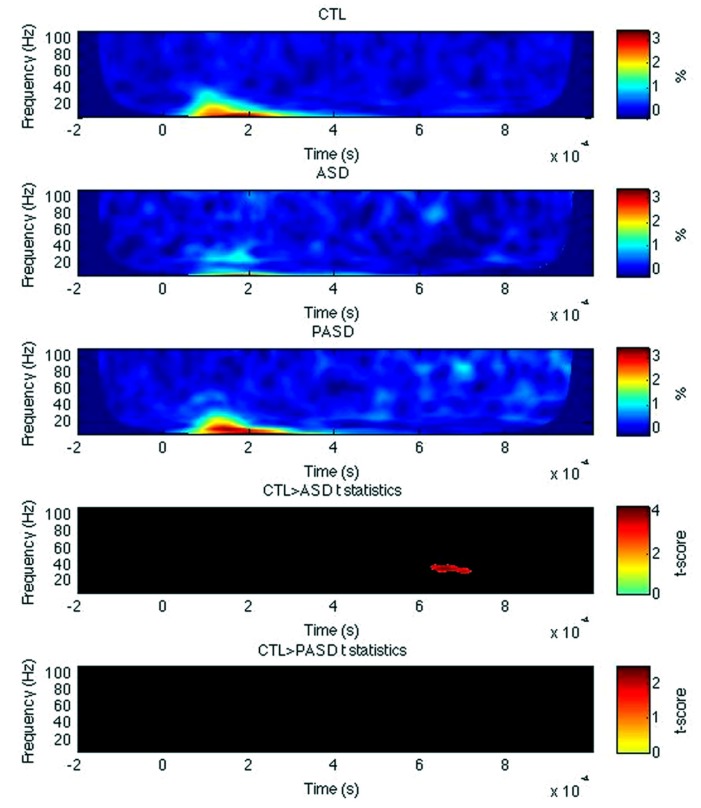
**Inferior frontal gyrus evoked power time-frequency results.** Grand average evoked power is shown for each group (three top rows) for the left inferior frontal gyrus (IFG). *T*-statistic maps are shown in the two bottom rows that illustrate contrasts between controls (CTL) and ASD (row 4) and CTL and parents (PASD; row 5). Masked *t*-score results represent cluster-corrected findings at *p* < 0.01.

For left IFG, there was a significant decrease in evoked power of the high beta/low gamma-band between 630 and 720 ms post-stimulus in the control group compared to the autism group (**Figure 4**). No other significant differences were observed, for any measures within the right IFG and for PLF or induced power in the left IFG.

#### Occipital lobe

**FIGURE 5:**
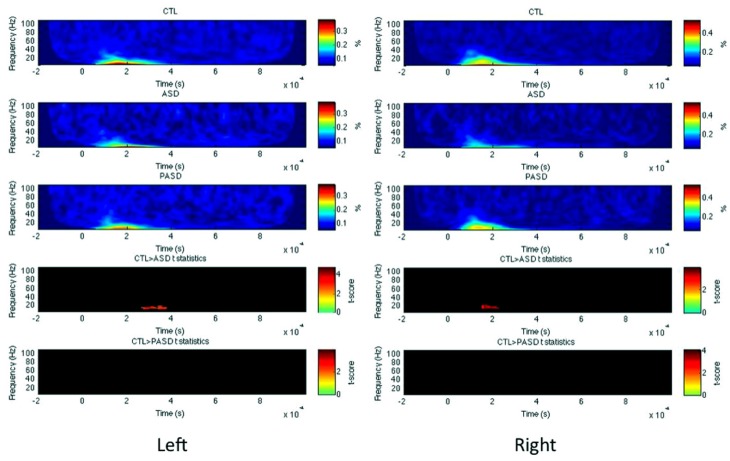
**Occipital lobe PLF time-frequency results.** Grand average PLF are shown for each group (three top rows) for the left and right occipital lobe (OCC). *T*-statistic maps are shown in the two bottom rows that illustrate contrasts between controls (CTL) and ASD (row 4) and CTL and parents (PASD; row 5). Masked *t*-score results represent cluster-corrected findings at *p* < 0.01.

In both left and right OCC (**Figure 5**), PLF but not evoked/induced power was significantly reduced in the ASD group in the beta band around 200 (for the right) and 300–400 ms (left). No difference between the parents and controls was found in any measures and any hemispheres.

### BEHAVIORAL RESULTS

**FIGURE 6:**
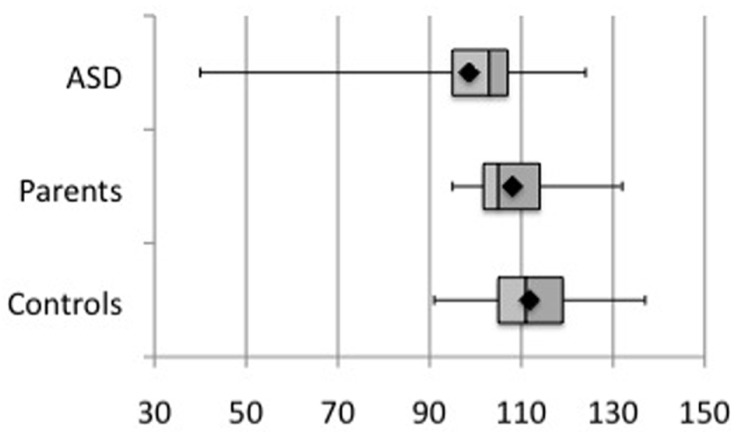
**Comparison of PPVT performance scores across group subjects.** No significant difference was observed (*p* = 0.50).

All participants were asked to complete the PPVT language test. **Figure 6** shows the scores for each group. Comparison between groups did not yield any statistical difference, according to a Kruskall–Wallis test (*p* = 0.50). There were no significant correlations between PPVT performance and either early or late high-gamma-band PLF, evoked or induced power, or those measures in the beta-band, even at uncorrected *p* < 0.05.

### ALTERED FUNCTIONAL CONNECTIVITY IN THE AUTISM GROUP

#### Left inferior frontal gyrus to left fusiform

**FIGURE 7:**
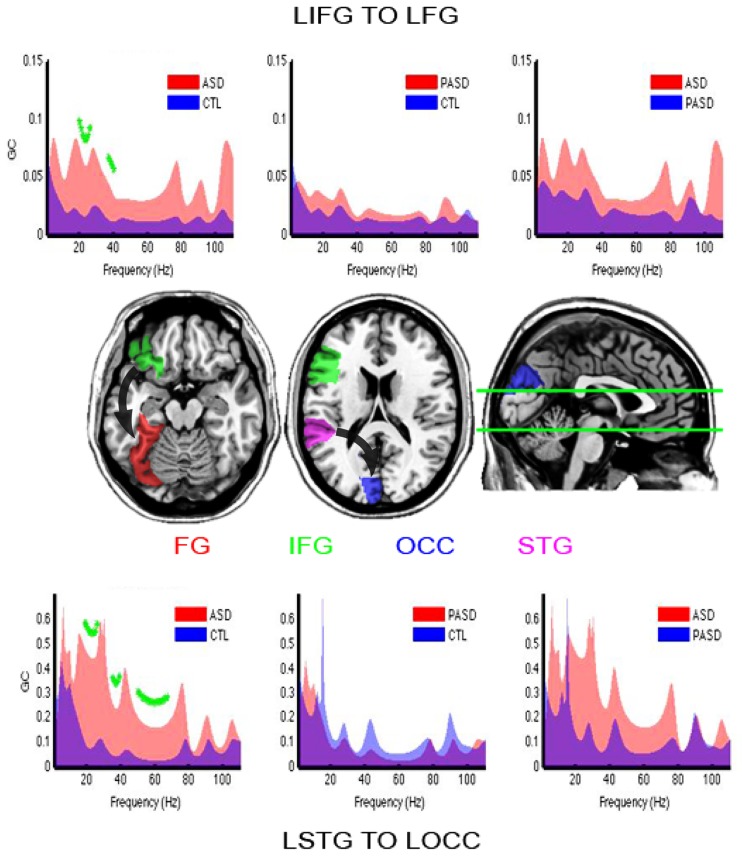
**Granger causality results.** Granger spectra show functional connectivity between regions of interest with significant differences between the ASD and control groups. GC, Granger causality; FG, fusiform gyrus; STG, superior temporal gyrus; IFG, inferior frontal gyrus; OCC, occipital lobe. The title reading from left to right (e.g., IFG–FG) indicates the direction of causation. Significant group differences are shown with green asterisks (FDR, *q* < 0.1). Brain slices in the middle row illustrate location of those brain areas and flux of information (arrows) between them.

Increased directional connectivity was observed between the left IFG to the left FG (**Figure 7**, top and horizontal slice) for the autism group compared to the control group. This increase was significant across the high-beta and low gamma frequencies after correction for multiple comparisons (FDR, *q* < 0.1).

#### Left superior temporal gyrus to left occipital lobe

As found between left IFG and FG, there was stronger connectivity in the group of autism participants compared to the controls from the left STG to the left OCC (**Figure 7**, bottom and right horizontal slice). This was statistically significant within the full beta-and gamma-band ranges.

No other significant differences in connectivity within or between hemispheres, for any frequency, were found among groups. We also observed no significant correlations between the connectivity data and the PPVT.

## DISCUSSION

Our results, if replicated, suggest that altered high- and low-frequency brain oscillations in regions involved in object and language-related processes are a notable characteristic of autism, and that first-degree relatives share some of those differences. The findings appeared to be regionally and temporally specific in the context of the current task and sample.

### CHANGES IN OSCILLATORY POWER AND OVER-CONNECTIVITY IN AUTISM

Converging evidence from across many studies and a variety of experimental paradigms suggests a gamma-band deficit in ASD (Grice et al., 2001; Brown et al., 2005; Wilson et al., 2007; Rojas et al., 2008, 2011; Gandal et al., 2010; McFadden et al., 2012; Wright et al., 2012; Gao et al., 2013), which has been proposed as a biomarker of autism (Uhlhaas et al., 2010).

In the STG, decreased high-gamma band activity was found. Using auditory-verbal stimulus material, we have already reported similar STG activation trends within autism (Wilson et al., 2007) albeit at a lower gamma-band frequency range of 30–50 Hz. In a language context, both structural and functional differences in the STG have been described in previous autism studies (Herbert et al., 2002; Harris et al., 2006; Hubbard et al., 2012). Altered gamma-band responses have also previously been reported in the STG of children with autism in response to simple, pure tone auditory stimulation (Gandal et al., 2010). In the left IFG, we also found decreased evoked power in the low-gamma band in autism compared to controls. Alterations in oscillations patterns in those language-network structures may be related to language impairments observed in people with autism.

We also note that the low gamma-band findings extended to the beta-range. Indeed, impaired beta-band oscillations have previously been observed in children with ASD compared to healthy controls (Stroganova et al., 2007, 2012). The delineation between the end of one band and beginning of the next is relatively arbitrary, so it is not unexpected to find that high beta and low gamma changes would be present. Additionally, beta-band oscillatory activity is independently known to be responsive to language stimuli (Hirata et al., 2004).

In the OCC, reduced beta PLF was found in both left and right sides of subjects with autism. The presence of those abnormalities in early visual responses is consistent with previous neurophysiological research on face processing (Dawson et al., 2005). Using MEG (Kylliainen et al., 2006), also reported differences in the processing of faces and other complex objects (motorbikes) at 100 ms in ASD children matched with typically developing controls. Those results and our study showing an impaired oscillation pattern related to object naming in autism suggest that visual brain activity may partly reflect general visual processing differences observed in this population (Jemel et al., 2006).

The observed changes in the STG and IFG occurred later in the post-stimulus window than the early phase-locked changes observed for the OCC. This is in accordance with the involvement of the occipital areas in visual functions, whereas the left FG (Binder et al., 1997; Balsamo et al., 2006) and the STG (Heath et al., 2012) have been reported as active during semantic processing at latter stages in the process of picture naming. A previous MEG study on picture naming reported visual and semantic processing around 0–150 and 275–400 ms after stimulus presentation (Levelt et al., 1998). Our differences were found between 600 and 900 ms, the timing of which suggests a role in early semantic or covert speech processes.

The autism group exhibited stronger functional connectivity from anterior to posterior language and visual areas compared with the control and parent groups, which may partially explain the impaired activation we found in that group in those regions. This finding is suggestive of differences in long-range neural synchronization present in our patient group. Alteration of long-range connectivity is an often reported finding in autism (Courchesne and Pierce, 2005). For example, reduced non-directional functional connectivity between anterior and posterior speech areas has been reported previously (Just et al., 2004). Our current result, however, does not support the underconnectivity theory. It should be noted that, apart from our finding of overconnectivity, other evidence also suggest overconnectivity in autism, such as a recent study showing enhanced functional excitation from occipital to frontal areas (Dominguez et al., 2013). In another recent paper, individuals with Asperger syndrome had higher, not lower, fractional anisotropy than controls in a diffusion tensor study of white matter (Roine et al., 2013). Together, these findings suggest that both underconnectivity and overconnectivity can be observed in autism relative to control subjects.

The range of oscillations where ASD people exhibited higher connectivity included both beta and gamma frequencies. Literature reports that gamma rhythms are prevalent in local visual response synchronization, but more distant coherence occurring during multimodal integration between parietal and temporal cortices uses rhythms in the beta range (Roelfsema et al., 1997). Since gamma-band activity is phase-amplitude coupled to lower frequency alpha- and theta-band oscillations, it is possible that the higher connectivity we observe in the gamma-range is a direct effect of the increased connectivity in the beta band between the same regions. A recent MEG paper reported that reduced high-gamma connectivity between the FG and other brain areas was related to decreased local connectivity, as assessed by phase-amplitude coupling to low frequency oscillations in those areas (Khan et al., 2013). One difference between this study and ours was the involvement of the FG relative to the task. In the paper reporting underconnectivity (Khan et al., 2013), the stimuli were faces; in ours, all of the stimuli were non-face objects. Thus, FG might be differentially affected in autism in a task specific manner.

The PPVT scores were not significantly different between the ASD and control groups. Together with the oscillatory and connectivity changes, this might suggest that the increase in communication between language and visual areas could provide a compensatory mechanism for coping with language and/or object naming difficulties. We also did not find any significant relationships between our groups’ performance on the behavioral language task and on their gamma-related results. This indicates that this paradigm might not well-suited to study language processes per se in autism, but is perhaps more relevant to issues in early visual perception and object recognition. Consistent with this, for example, a previous study reported FG activation during color naming (Price and Devlin, 2003). Finally, we note that although we have speculated concerning language deficits in autism, there is no behavioral evidence for such deficits in our sample. A more severely affected sample might be necessary to establish a relationship, if any, between oscillatory power changes, connectivity, and language deficits in people with autism.

### A POTENTIAL COMPENSATORY/PROTECTIVE MECHANISM IN FIRST-DEGREE RELATIVES

In the FG, evoked power revealed an increase in late high-beta/low-gamma band activity in the PASD group compared to controls. Similarly, we recently published evidence of gamma-band over-activation in the FG in autism first-degree relatives using language stimuli delivered in the auditory modality (McFadden et al., 2012). This increase also extended to the beta-band. Indeed, parents of children with autism show evidence of several areas of difference in common with persons with ASD, such as face memory and object recognition (Wallace et al., 2010), social cognition and working memory (Gokcen et al., 2009), and executive function, the latter being also shared by unaffected siblings (Wong et al., 2006). At the functional level, our group has recently published the evidence of differences in neural patterns associated with phonological processing in first-degree relatives (Wilson et al., 2012).

In the STG, increased activation in the high-gamma band was found in the left hemisphere of parents. Here again, we have already reported similar STG activation trends within autism relatives (McFadden et al., 2012) albeit at a lower gamma-band frequency range of 30–50 Hz. This finding is opposite to what we found in the ASD group. Such differential findings have been reported previously in autism subjects and relatives, such as intact verbal IQ in relatives while probands generally exhibit lower levels relative to performance IQ (Schmidt et al., 2008). In this context, it is possible that the observed over-activity in language-related regions is either a compensatory or protective mechanism.

## CONCLUSION

Our findings of altered beta and gamma oscillations in people with ASD is consistent with a change in neural synchrony, which adds to a growing literature on gamma-band deficits across a number of simple sensory and complex cognitive tasks. The findings suggest that such oscillatory changes may also be relevant to higher order visual object processing and possibly to some language functions, at least in the context of object naming. The lack of similarity between the probands and the parents represents a challenge to the endophenotype interpretation. Alternative explanations include a compensatory or protective mechanism in the first-degree relatives. Impaired connections between posterior and anterior regions of the brain may be a marker of language and/or visual processing differences in autism, but future studies of language impaired individuals with autism will be needed to clarify a specific role, if any, for altered intra-hemispheric connectivity in the language processing deficits observed in the disorder.

## Conflict of Interest Statement

The authors declare that the research was conducted in the absence of any commercial or financial relationships that could be construed as a potential conflict of interest.

## Footnotes

1

2
